# A fragmented segment of a central venous catheter caused delayed ventricular fibrillation: a case report

**DOI:** 10.1186/s40981-023-00615-x

**Published:** 2023-05-17

**Authors:** Kei Takahashi, Takero Arai, Takashi Asai, Yasuhisa Okuda

**Affiliations:** Department of Anesthesiology, Dokkyo Medical University Saitama Medical Center, 2-1-50 Minami-Koshigaya, Koshigaya City, Saitama, 343-8555 Japan

**Keywords:** Ventricular fibrillation, Cardiopulmonary resuscitation, Central venous access port

## Abstract

**Background:**

Central venous port systems may be safely used for chemotherapy of patients with cancer, but several complications may occur associated with their use.

**Case presentation:**

An 83-year-old man with heat stroke was transferred to our emergency department, where he was treated and became able to eat on the same day. He had been fit and healthy, except for colorectomy and chemotherapy using a central venous access port placed in the right upper jugular vein 8 years ago. The next day, he suddenly had ventricular fibrillation. Cardiopulmonary resuscitation was successful. Emergency coronary angiography showed a catheter-like foreign body in the coronary sinus. Physicians failed to remove the foreign body using catheter therapy, and ventricular fibrillation occurred repeatedly. After induction of general anesthesia, the fractured catheter was removed surgically. Postoperative course was uneventful.

**Conclusions:**

A fragmented segment of a catheter may suddenly cause ventricular fibrillation years later.

## Background

Central venous port systems may be safely used for chemotherapy of patients with cancer, but complications may occur in 7–16% of cases [1, 2]. Complications during insertion of the device (or an ordinary central venous catheter) include venous malposition, bleeding, vascular injury, pneumothorax, hemothorax, cardiac tamponade, and fracture of a catheter or a stylet. Delayed complications associated with the use of a central venous port system include port camber rotation, thrombosis, infection, catheter pinch-off syndrome, and spontaneous fragmentation of a catheter [1, 2].

Spontaneous fragmentation of a catheter may become a foreign body in the right atrium, the right ventricle, the pulmonary artery [2, 3], and in rare cases, in the coronary sinus [4–7]. In the reported cases of the foreign body in the coronary sinus [4–7], no apparent symptoms were provoked and were accidentally found by imaging, such as radiography. The foreign body was removed either by a percutaneous loop-snare technique or by open thoracotomy.

This is the first report of ventricular fibrillation which was likely to be caused by a fractured catheter located in the coronary sinus and first report of general anesthesia to a patient with such a complication.

## Case presentation

We obtained a verbal informed consent from the patient and a written informed consent from a family member of the patient for publication of this case.

An 83-year-old man was transported to our emergency department, with a tentative diagnosis of heat stroke. He had previously been fit and healthy, except for undergoing colorectomy for colorectal cancer 8 years ago. He also received a central venous port system, a BardPort Titanium implantable port with its and 8.0 Fr Groshong® silicone catheter (Bard Access Systems, Inc., Salt Lake, USA) which was inserted through the right internal jugular vein, for chemotherapy.

He was transferred to the intensive care unit, where surface cooling and intravenous hydration were performed to treat heat stroke. He made a rapid recovery, and he had become able to eat on the same day.

**Fig. 1:**
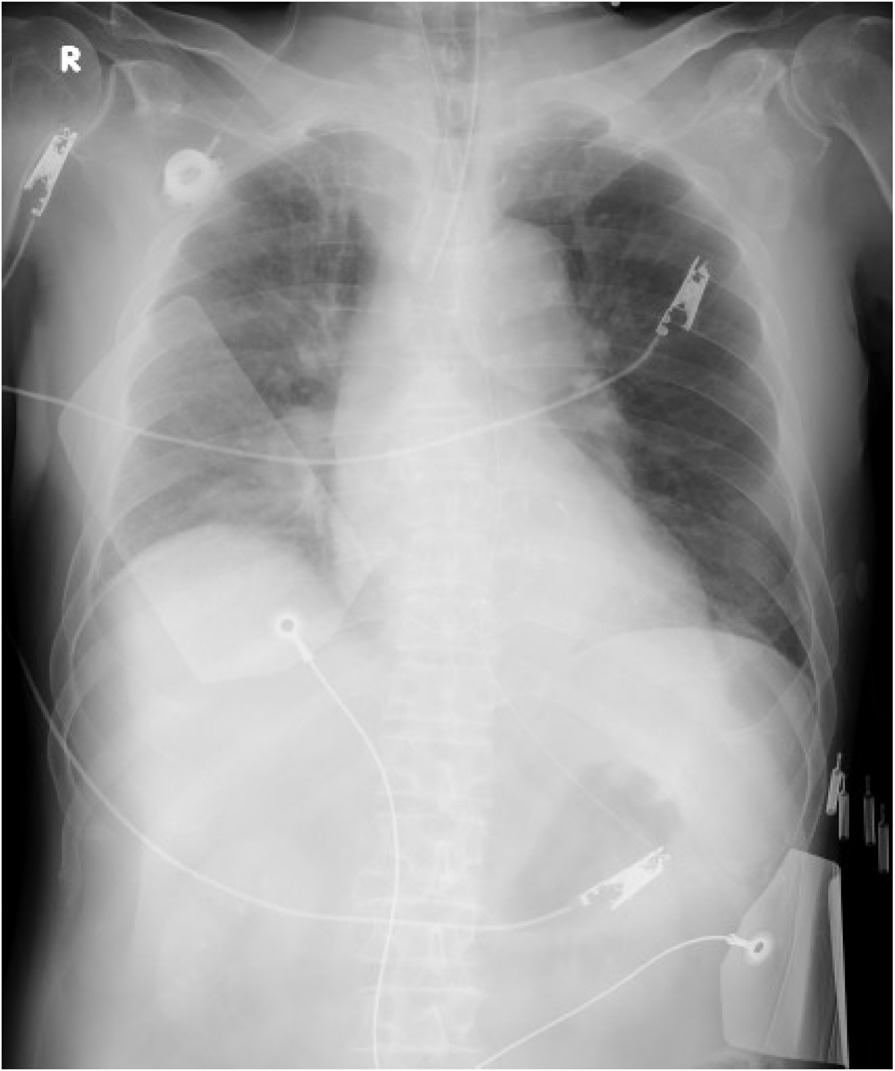
Chest X-ray, taken immediately after cardiopulmonary resuscitation, of a patient with heat stroke and cardiac arrest, indicated no apparent cardiopulmonary abnormalities

Next morning, he suddenly developed ventricular fibrillation. Cardiopulmonary resuscitation was performed immediately, his trachea was intubated, and he was sedated with continuous infusion of propofol. He had not complained any chest pain before the occurrence of ventricular fibrillation, and the electrocardiography indicated neither arrythmias nor ST changes, before and after the occurrence of ventricular fibrillation. Analysis of arterial blood taken after resuscitation indicated no abnormalities in the blood gases and the blood electrolyte; troponin was not measured. A chest radiograph, which was taken after resuscitation (Fig. 1), indicated a previously placed central venous catheter access port, without any obvious abnormalities.

Emergency coronary angiography, performed shortly after ventricular fibrillation, identified a 90% stenosis of the left anterior descending coronary artery. The cardiologists considered that this was not the direct cause of ventricular fibrillation, as the extent to which the blood flow in the coronary artery would not bring about ventricular fibrillation. The angiography instead identified a more likely cause: a foreign body in the coronary sinus.

**Fig. 2:**
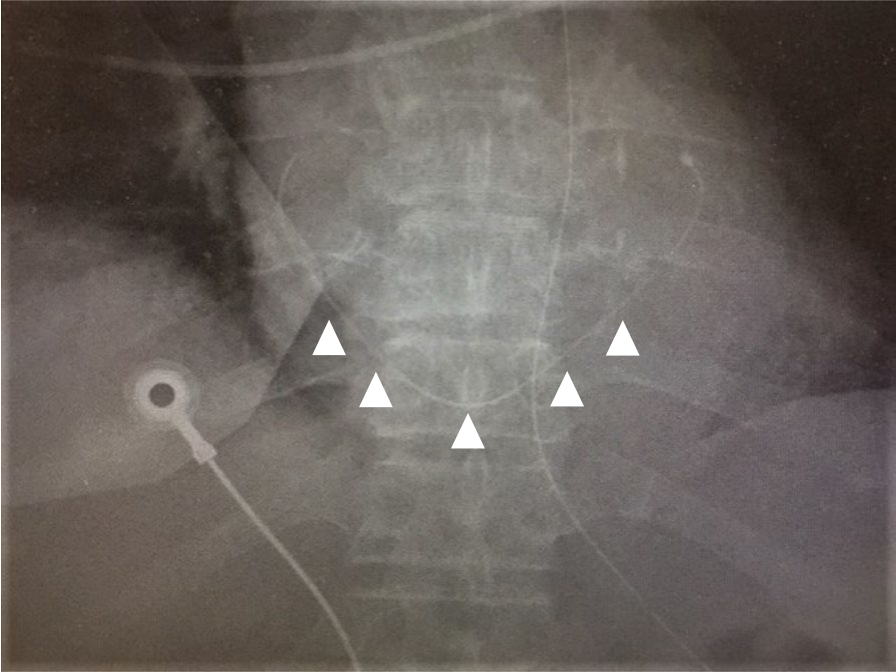
A close examination of the chest X-ray (Fig. 1) taken immediately after cardiopulmonary resuscitation indicated a severed catheter (arrows)

**Fig. 3:**
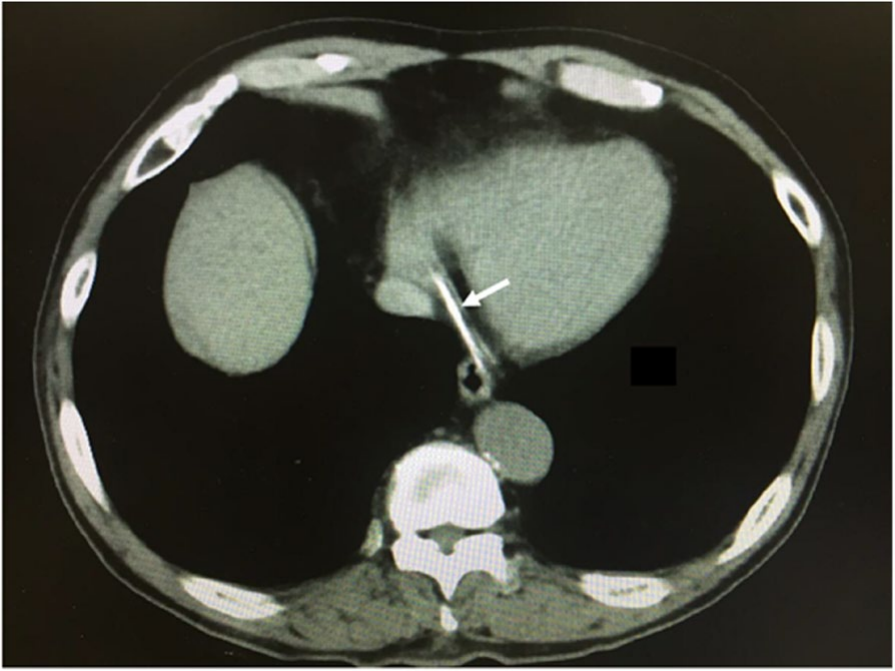
The computed tomography confirmed the presence of a catheter-like foreign body in the coronary sinus and the great cardiac vein (arrows)

Close re-examination of the chest radiograph taken after resuscitation showed a long catheter-like foreign body (approximately 20 cm) in the heart (Figs. 1 and 2). Chest computed tomography also confirmed the presence of the foreign body (Fig. 3). We diagnosed that a long catheter-like foreign body, likely to be a fragmented central venous catheter, was in the coronary sinus.

In the angiography suite, physicians attempted to remove the foreign body. However, the attempts were abandoned, as the pull of the catheter resulted in repeated ventricular fibrillation. The patient was successfully resuscitated. Surgical removal was planned the next day.

On the next day, the patient was transferred to an operating room, and routine monitors, such as a pulse oximeter, a blood pressure cuff, and electrocardiogram, were attached. We prepared an electric defibrillator, and cardiac surgeons were present in the room. The breathing system of an anesthesia machine was connected to the tracheal tube, and general anesthesia was induced with sevoflurane 1.5% and fentanyl 0.1 mg; neuromuscular blockade was achieved with rocuronium 50 mg. Anesthesia was maintained with sevoflurane and oxygen in air.

**Fig. 4:**
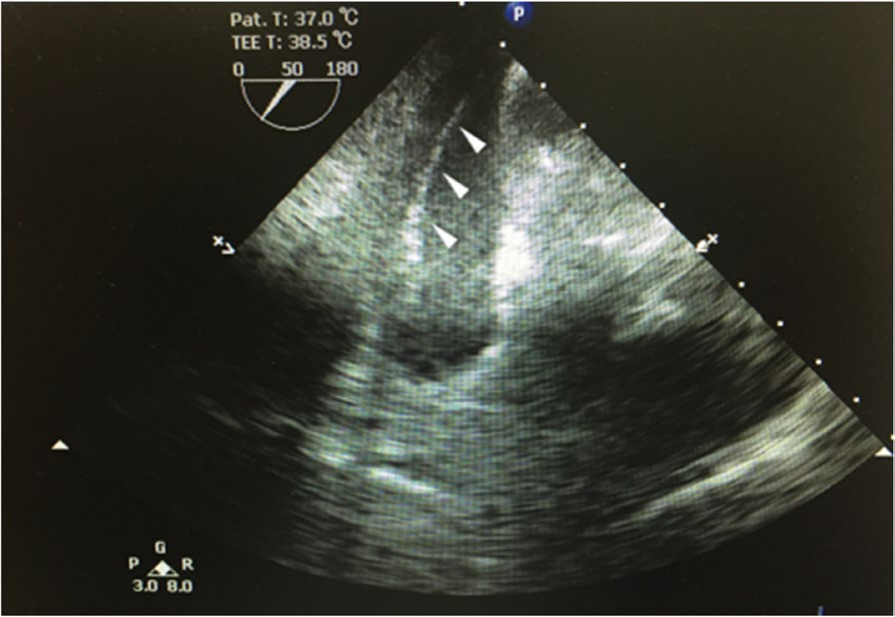
The fractured catheter was identified into coronary sinus by intraoperative transesophageal echocardiography (arrows)

**Fig. 5:**
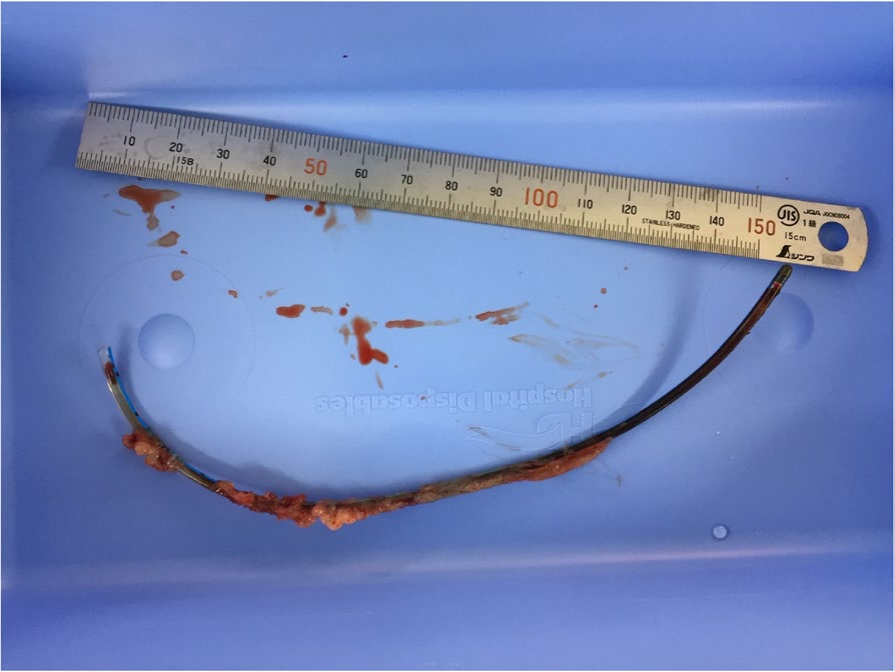
A severed catheter (an 8.0-Fr Groshong silicone Catheter), which was connected to a BardPort Titanium implantable port (Bard Access Systems, Inc., Salt Lake, USA))

The patient underwent sternotomy, and transesophageal echocardiography identified the fractured catheter in the coronary sinus (Fig. 4). The right atrium was opened under cardiopulmonary bypass. Surgeons could remove the fractured catheter (approximately 20 cm in length) (Fig. 5), but with great difficulty, by gently pulling the edge of the catheter with rotating movement. After the operation, the electrocardiogram indicated no arrhythmia or cardiac ischemia.

On the next day of the operation, his family requested strongly to treat the coronary stenosis, and thus, cardiac physicians placed a stent in the left anterior descending artery without complications. He was discharged from the hospital on postoperative day 14, to a rehabilitation facility, and he went home at a later day.

## Discussion

In our patient, it was initially difficult to identify the cause of ventricular fibrillation. One possible cause was 90% stenosis of the left anterior descending artery, but we judged that was not the likely cause, because of the following reasons. First, the extent to which the blood flow in the coronary artery would not bring about ventricular fibrillation. Second, the patient did not complain any chest pain before the occurrence of ventricular fibrillation, and the electrocardiogram indicated normal heart rhythm without ST changes, before and after recovery from the ventricular fibrillation (although myocardial infarction without chest pain or ST change may occur) [8]. Third, ventricular fibrillation occurred when cardiologists tried to remove the fractured catheter, and no ventricular fibrillation or ST changes occurred after removing of the fractured catheter. Because of these reasons, the fractured catheter in the coronary artery was a more likely cause of ventricular fibrillation.

It is not clear when and how the catheter was severed and migrated from the vena cava to the right atrium and then retrogradely to the coronary sinus. The chest X-ray taken after insertion of the central venous catheter confirmed the correct position in the superior vena cava. We speculate that, because the fractured catheter was strongly adherent to the coronary sinus (Fig. 2), it is likely to the catheter had been in the coronary sinus for a long time without causing any problem. There have been reports of a spontaneously fragmented catheter migrated to the coronary sinus (with unknown mechanism) [4–7], and similar to our case, no apparent symptoms were evoked by the foreign body.

The exact cause of ventricular fibrillation which occurred 8 years later from inserting central venous port systems is also not known. The most likely reason is previous day’s dehydration due to heat stroke: dehydration might have reduced the coronary blood flow, massive fluid treatment at the hospital would have made his heart bigger (compressing the coronary arteries), and the fractured catheter might have stimulated the heart tissue, leading to ventricular fibrillation.

In conclusion, we have experienced general anesthesia to a patient with repeated ventricular fibrillation due to a fractured catheter in the coronary sinus. A fragmented segment of a catheter may suddenly cause ventricular fibrillation years later. Anesthesiologists, surgeons, and all the medical staff in the operation room should cooperate and take immediate action if ventricular fibrillation occurs perioperatively.
